# Top 100 Most Cited Articles on Intraoperative Image-Guided Navigation in Spine Surgery

**DOI:** 10.7759/cureus.67950

**Published:** 2024-08-27

**Authors:** Fernando González-González, Felipe Aguilar-Chávez, Carolina Martínez-Loya, Luis A Marín-Castañeda, Carlos A Arellanes-Chavez, Ángel Lee

**Affiliations:** 1 Cisne Spine Academy, Star Medica Hospital, Autonomous University of Chihuahua, Chihuahua, MEX; 2 Research, Faculty of Medicine and Biomedical Sciences, Autonomous University of Chihuahua, Chihuahua, MEX; 3 Neurology, La Salle University College of Medicine, Mexico City, MEX; 4 Research, Dr. Manuel Gea González General Hospital, Mexico City, MEX

**Keywords:** citation analysis, bibliometric, neuronavigation, computer-assisted, spine surgery

## Abstract

Navigation technologies have become essential in spine surgery over the last decade, offering precise procedures and minimizing risks. To the best of our knowledge, this is the first bibliometric analysis on this topic, providing insights and trends on topics, authors, and journals. The study identifies and analyzes the 100 most cited articles related to navigation in spine surgery.

A systematic search was performed in Scopus and Google Scholar to identify all articles related to navigation in spine surgery (38,057 articles). The 100 most cited were analyzed for citations, titles, abstracts, authors, affiliations, keywords, country and institute of origin, year of publication, and level of evidence. The search was conducted in October 2023.

The 100 most cited articles were published between 1995 and 2019, with 2010 to 2019 being the most prolific decade (46%). The most cited article had 733 citations, and the paper with the most citations per year averaged 59.27 citations/year. The *Spine Journal* had the most articles (34%). The United States contributed the most articles (39%). Most publications were clinical research and reviews (94%), with an overall evidence grade of IV-V (63%). A positive trend was noted in the last decade for incorporating augmented reality.

This bibliometric analysis offers valuable insights and trends in spine surgery navigation literature. The findings indicate that technological advancements have led to more articles with higher levels of evidence. These pivotal articles shape evidence-based medicine, future surgeons, and industry improvements in navigated spine surgery.

## Introduction and background

Navigation for spine surgery was developed more than 30 years ago; in the last decade, it has become a paramount tool that empowers spine surgeons with real-time, three-dimensional guidance, enabling them to sail through complex anatomical structures with confidence and accuracy [1]. Whether addressing spinal deformities, tumors, fractures, or degenerative conditions, navigation technologies can provide more precise placement of material such as screws, cages, or spacers while minimizing risks associated with the procedure [2,3].

The bibliometric analysis is a quantitative method used by scholars to review in-depth scientific literature [4]. It involves the analysis of bibliographic data, such as citations, publication records, and authorship information to highlight trends, research gaps, and patterns within a specific academic area [5]. It is important to note that this type of method offers information such as research output and publication impact that can ultimately benefit academics and alter their practice within the field [4].

Literature analysis requires a thorough assessment of the publications to identify influential authors, journals, and trends, aiding researchers, institutions, and policymakers in making informed decisions [6]. The impact factor of a journal is directly influenced by the number of citations received for each article published within a determined period of time [7].

In recent years, there have been several publications examining the most influential articles published within the spine surgery field, specifically cervical spine, lumbar spine, deformity surgery, and minimally invasive surgery, but not on navigation for spine surgery [8,9]. This is the first publication to enlist the most influential articles relevant to spine surgery assisted with navigation. The aim of this study is to identify the top 100 most cited papers in this field and serve as a guide for surgeons, fellows, and residents to understand and adopt this technology.

## Review

This study was exempt from institutional review board approval. Since there is no consensus on a guideline for bibliometric and related analyses (The Guidance List for the repOrting of Bibliometric AnaLyses, GLOBAL, pending publication). The Preferred Reporting Guidelines for Systematic Review (PRISMA) 2020 checklist was followed to ensure a transparent and complete report of our findings. On August 11, 2023, a primary search was performed in Scopus and then Google Scholar was used as a secondary retrieval tool, as they both encompass the biggest journal databases, with the latter being more unrestrictive on the information published. A search strategy was established to obtain the largest number of articles related to navigated spine surgery. The following terms were used: ((“Spine” or “Spine surgery”) AND (“Navigation” OR “neuronavigation” OR “3D-fluoroscopy” OR “3D-navigation” OR “computer-assisted” OR “O-arm”)).

**Figure 1:**
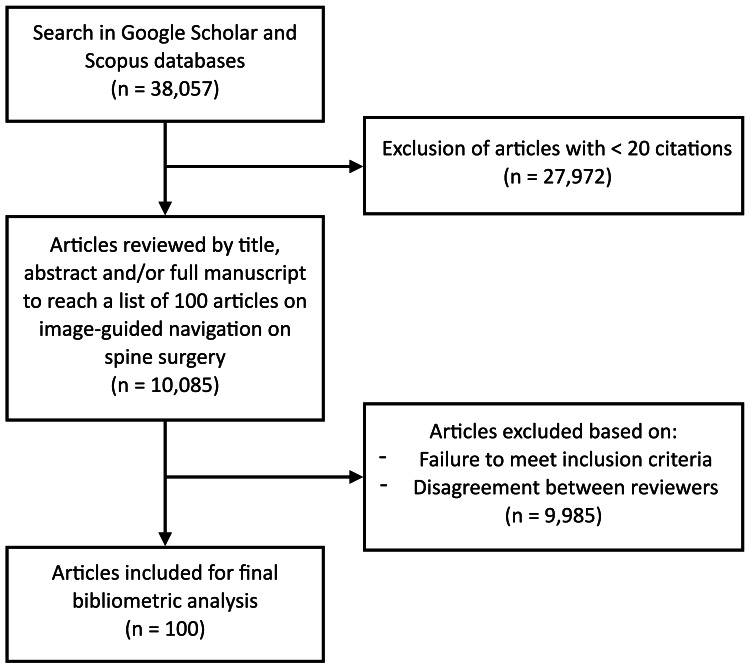
Flowchart of the screening process for relevant articles

The search criteria and filters were restricted to peer-reviewed articles and review articles, excluding all other publications such as letters, notes, books, and conference papers. Both medical and non-medical journals were included. Only articles in English and Spanish were included. We did not impose restrictions on the publication date or subject/research area. No restrictions were placed on the study type, authors, editors, affiliation, funding or sponsor company, country/territory of origin, or source type. The 38,057 resulting articles were then ranked by the number of citations, in descending order. Articles with fewer than 20 citations were excluded (27,972 articles), and 10,085 documents were exported in CSV format and organized using the MacOS Numbers (Apple, Inc., Cupertino, CA, USA) software application for analysis of the title, abstract, and/or full manuscript until a list of 100 articles was reached (Figure 1).

The list of articles was subjected to a strict selection process; initially, all titles and abstracts relevant to image-guided navigation in spine surgery were highlighted. Subsequently, the document's abstract was reviewed by three independent authors (C.A.A.C., C.M.L., and F.A.C.) and full manuscripts were evaluated if necessary to verify if the article met the inclusion criteria. Articles in question for inclusion were reviewed independently by the lead author (F.G.G.). The screening stopped when 100 documents were found. We included articles pertinent to navigation in spine surgery, based on one or more of the following criteria: (1) navigated instrumentation of the spine (occipital, cervical, thoracic, lumbar, sacral, pelvic); (2) navigated use of spacers, cages or other implants of the spine; (3) navigated surgery of the spine pertaining degenerative, deformity, traumatic o tumoral pathology; (4) any type/brand of intraoperative navigation system for spine surgery; (5) spine surgery with navigation for in vivo, cadaveric and in vitro models; (6) navigated spine surgery used as the comparative or main topic of the article. We excluded articles that did not focus on navigation for spine surgery. The articles included were accepted by all the authors, if an article was not considered as suitable by one of them, it was excluded.

The final list of the 100 most cited articles was then selected and assessed for the number of citations, title, authors, journal, country and institution of origin, year of publication, and level of evidence (LOE). The complete manuscript was analyzed, and information relevant to statistical and demographic analysis was gathered; information was cross-referenced with Scopus, PubMed, and Google Scholar. Lastly, the LOE I-VII was assigned by all authors based on the manuscript methods. On October 2, 2023, a last search was done to ratify any changes in the citation number records for the included articles.

Results

**Figure 2:**
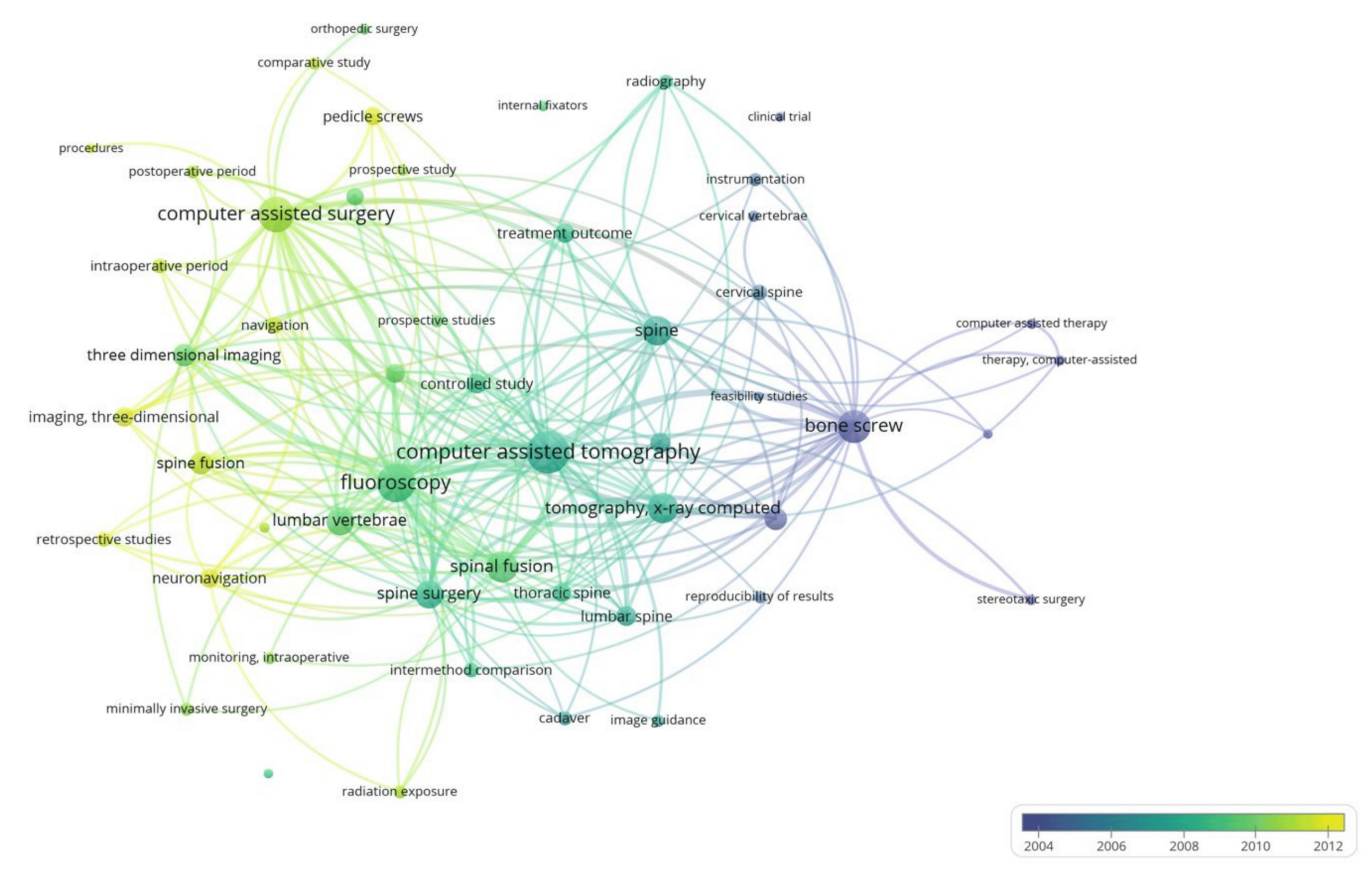
Keyword relationship between articles, stratified by year clusters This figure illustrates the network visualization of main keywords, created using VOS Viewer. Each keyword is represented by a circle, where the size of the circle corresponds to the keyword frequency; a larger circle means the keyword appears with more frequency in the articles. The lines connecting the circles indicate the strength of the association between keywords; the more lines attached to a keyword mean it is more often used in combination with other connected keywords. Additionally, the color of the circles and lines reflects the temporal evolution of the keywords over the last years, with different colors representing different years, allowing for an analysis of how the focus of research has shifted over time.

The top 100 most cited articles for navigation in spine surgery were published between 1995 and 2019 (Table 1). The most prolific decade was from 2010 to 2019 with 49 articles; a positive trend in publications was identified since the decade from 2000 to 2009 with 40 articles and from 1990 to 1999 with 11 articles. All articles were found to be written in English. Figure 2 shows the strength of co-occurrence between keywords used in the analyzed papers.

**Table 1:** General information related to the 100 articles included

Description	Results
Year of publication	1995-2019
Journals	28
Mean article's tenure	14.9 years
Mean citations per document	201.84
Number of authors	469
Co-authors per document	5.97
Single-authored documents	0
International co-authorships	17.51%
Keywords	621
Author’s keywords	208
References	2453

**Figure 3:**
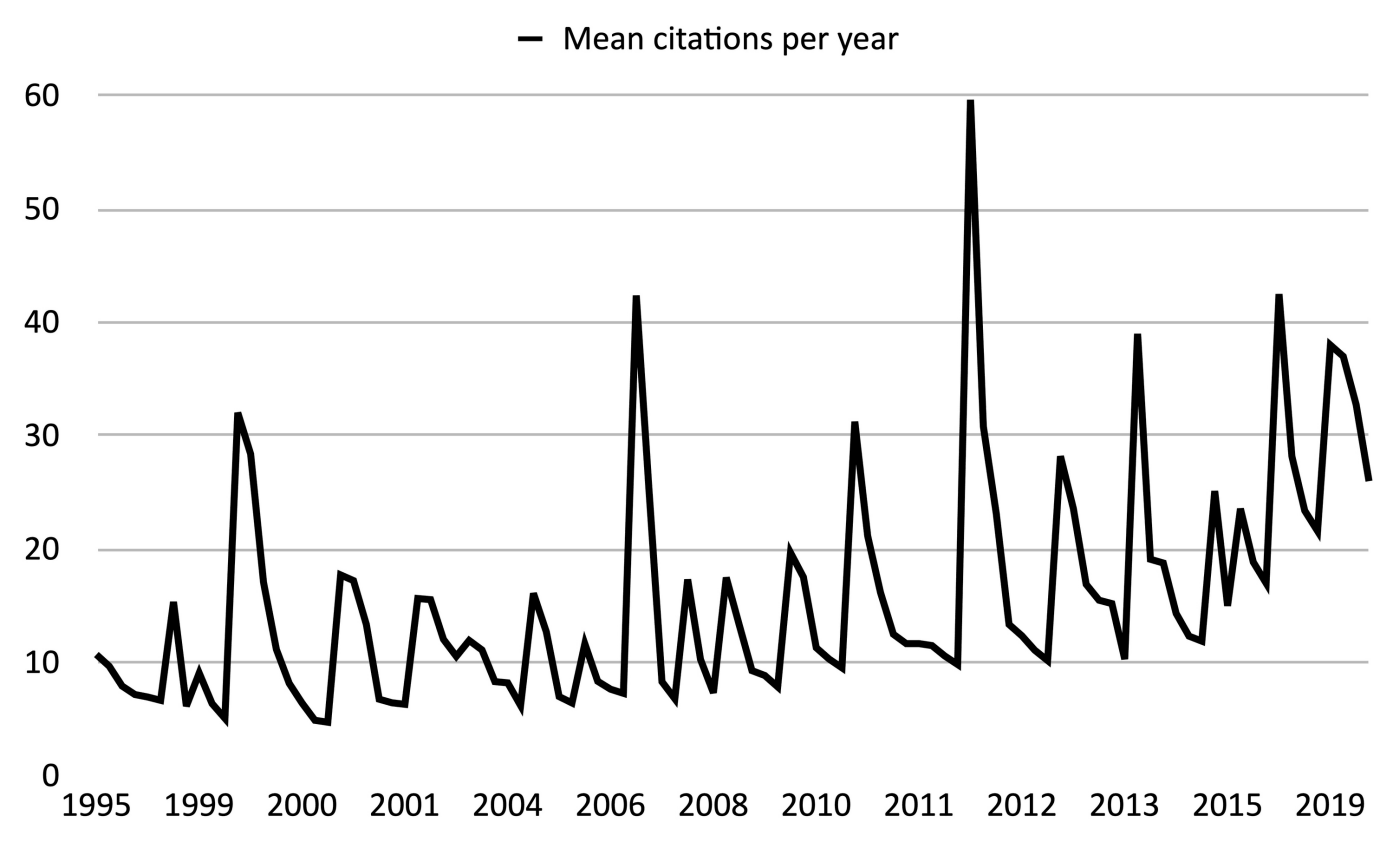
The graph illustrates the mean number of citations received by the top 100 cited articles between 1995 and 2019.

The combined number of citations was found to be 20,184; the most cited paper had 737 citations while the least cited paper had 76 citations, and the mean was 201.84 citations per article. The paper with the most citations per year obtained 59.27, while the article with the least citations per year had 4.74; the mean was 15.60 citations/year per article (Table 2). An increase in mean citations per year was observed for articles from recent years, compared to papers from the 90s (Figure 3).

**Table 2:** List of the top 100 cited articles in navigation for spinal surgery * indicates the number of citations based on Scopus.

Rank	Articles	Evidence level	Citations*	Citations per year
1	Laine T, Lund T, Ylikoski M, Lohikoski J, Schlenzka D. Accuracy of pedicle screw insertion with and without computer assistance: a randomised controlled clinical study in 100 consecutive patients. Eur Spine J. 2000;9(3):235-40 [10].	1	733	31.87
2	Kosmopoulos V, Schizas C. Pedicle screw placement accuracy: A meta-analysis. Spine (Phila Pa 1976). 2007;32(3):E111-20 [11].	1	671	41.94
3	Gelalis ID, Paschos NK, Pakos EE, Politis AN, Arnaoutoglou CM, Karageorgos AC, et al. Accuracy of pedicle screw placement: a systematic review of prospective in vivo studies comparing free hand, fluoroscopy guidance and navigation techniques. Eur Spine J. 2012;21(2):247-55 [12].	1	652	59.27
4	Amiot L-P, Lang K, Putzier M, Zippel H, Labelle H. Comparative results between conventional and computer-assisted pedicle screw installation in the thoracic, lumbar, and sacral spine. Spine (Phila Pa 1976). 2000;25(5):606-14 [13].	3	649	28.22
5	Rajasekaran S, Vidyadhara S, Ramesh P, Shetty AP. Randomized clinical study to compare the accuracy of navigated and non-navigated thoracic pedicle screws in deformity correction surgeries. Spine (Phila Pa 1976). 2007;32(2):E56-64 [14].	1	397	24.81
6	Laine T, Schlenzka D, Mäkitalo K, Tallroth K, Nolte L-P, Visarius H. Improved accuracy of pedicle screw insertion with computer-assisted surgery: A prospective clinical trial of 30 patients. Spine (Phila Pa 1976). 1997;22(11):1254-8 [15].	1	397	15.27
7	Ludwig SC, Kramer DL, Balderston RA, Vaccaro AR, Foley KF, Albert TJ. Placement of pedicle screws in the human cadaveric cervical spine: Comparative accuracy of three techniques. Spine (Phila Pa 1976). 2000;25(13):1655-67 [16].	5	393	17.09
8	Foley KT, Simon DA, Rampersaud YR. Virtual fluoroscopy: Computer-assisted fluoroscopic navigation. Spine (Phila Pa 1976). 2001;26(4):347-51 [17].	5	390	17.73
9	Youkilis AS, Quint DJ, McGillicuddy JE, Papadopoulos SM. Stereotactic navigation for placement of pedicle screws in the thoracic spine. Neurosurgery. 2001;48(4):771-9 [18].	4	377	17.14
10	Tian N-F, Huang Q-S, Zhou P, Zhou Y, Wu R-K, Lou Y, et al. Pedicle screw insertion accuracy with different assisted methods: a systematic review and meta-analysis of comparative studies. Eur Spine J. 2011;20(6):846-59 [19].	1	375	31.25
11	Mason A, Paulsen R, Babuska JM, Rajpal S, Burneikiene S, Nelson EL, et al. The accuracy of pedicle screw placement using intraoperative image guidance systems: A systematic review. J Neurosurg Spine. 2014;20(2):196-203 [20].	1	349	38.78
12	Shin BJ, James AR, Njoku IU, Härtl R. Pedicle screw navigation: a systematic review and meta-analysis of perforation risk for computer-navigated versus freehand insertion: A review. J Neurosurg Spine. 2012;17(2):113-22 [21].	1	339	30.82
13	Kotani Y, Abumi K, Ito M, Minami A. Improved accuracy of computer-assisted cervical pedicle screw insertion. J Neurosurg Spine. 2003;99(3):257-63 [22].	4	313	15.65
14	Holly LT, Foley KT. Intraoperative spinal navigation. Spine (Phila Pa 1976). 2003;28(supplement):S54-61 [23].	5	311	15.55
15	Nolte L-P, Zamorano LJ, Jiang Z, Wang Q, Langlotz F, Berlemann U. Image-guided insertion of transpedicular screws: A laboratory set-up. Spine (Phila Pa 1976). 1995;20(supplement):497-500 [24].	5	299	10.68
16	Richter M, Cakir B, Schmidt R. Cervical pedicle screws: Conventional versus computer-assisted placement of cannulated screws. Spine (Phila Pa 1976). 2005;30(20):2280-7 [25].	2	289	16.06
17	Roser F, Tatagiba M, Maier G. Spinal robotics: Current applications and future perspectives. Neurosurgery. 2013;72(Supplement 1):A12-8 [26].	5	281	28.10
18	Lavallée S, Sautot P, Troccaz J, Cinquin P, Merloz P. Computer-assisted spine surgery:A technique for accurate transpedicular screw fixation using CT data and a 3-D optical localizer. Comput Aided Surg. 1995;1(1):65-73 [27].	5	271	9.68
19	Kim CW, Lee Y-P, Taylor W, Oygar A, Kim WK. Use of navigation-assisted fluoroscopy to decrease radiation exposure during minimally invasive spine surgery. Spine J. 2008;8(4):584-90 [28].	4	260	17.33
20	Rampersaud YR, Pik JHT, Salonen D, Farooq S. Clinical accuracy of fluoroscopic computer-assisted pedicle screw fixation: A CT analysis. Spine (Phila Pa 1976). 2005;30(7):E183-90 [29].	4	257	14.28
21	Verma R, Krishan S, Haendlmayer K, Mohsen A. Functional outcome of computer-assisted spinal pedicle screw placement: a systematic review and meta-analysis of 23 studies including 5,992 pedicle screws. Eur Spine J. 2010;19(3):370-5 [30].	1	255	19.62
22	Van de Kelft E, Costa F, Van der Planken D, Schils F. A prospective multicenter registry on the accuracy of pedicle screw placement in the thoracic, lumbar, and sacral levels with the use of the O-arm imaging system and StealthStation navigation. Spine (Phila Pa 1976). 2012;37(25):E1580-7 [31].	1	253	23.00
23	Overley SC, Cho SK, Mehta AI, Arnold PM. Navigation and robotics in spinal surgery: Where are we now? Neurosurgery. 2017;80(3S):S86-99 [32].	5	252	42.00
24	Silbermann J, Riese F, Allam Y, Reichert T, Koeppert H, Gutberlet M. Computer tomography assessment of pedicle screw placement in lumbar and sacral spine: comparison between free-hand and O-arm based navigation techniques. Eur Spine J. 2011;20(6):875-81 [33].	2	248	20.67
25	Tian N-F, Xu H-Z. Image-guided pedicle screw insertion accuracy: a meta-analysis. Int Orthop. 2009;33(4):895-903 [34].	1	244	17.43
26	Mirza SK, Wiggins GC, Kuntz C IV, York JE, Bellabarba C, Knonodi MA, et al. Accuracy of thoracic vertebral body screw placement using standard fluoroscopy, fluoroscopic image guidance, and computed tomographic image guidance: A cadaver study. Spine (Phila Pa 1976). 2003;28(4):402-13 [35].	5	239	11.95
27	Härtl R, Lam KS, Wang J, Korge A, Kandziora F, Audigé L. Worldwide survey on the use of navigation in spine surgery. World Neurosurg. 2013;79(1):162-72 [36].	5	232	23.20
28	Tjardes T, Shafizadeh S, Rixen D, Paffrath T, Bouillon B, Steinhausen ES, et al. Image-guided spine surgery: state of the art and future directions. Eur Spine J. 2010;19(1):25-45 [37].	5	228	17.54
29	Richter M, Mattes T, Cakir B. Computer-assisted posterior instrumentation of the cervical and cervico-thoracic spine. Eur Spine J. 2004;13(1):50-9 [38].	4	227	11.95
30	Amiot L-P, Labelle H, DeGuise JA, Sati M, Brodeur P, Rivard C-H. Computer-assisted pedicle screw fixation- A feasibility study. Spine (Phila Pa 1976). 1995;20(10):1208-12 [39].	5	222	7.93
31	Slomczykowski M, Roberto M, Schneeberger P, Ozdoba C, Vock P. Radiation dose for pedicle screw insertion: Fluoroscopic method versus computer-assisted surgery. Spine (Phila Pa 1976). 1999;24(10):975-83 [40].	2	214	8.92
32	Holly LT, Foley KT. Three-dimensional fluoroscopy-guided percutaneous thoracolumbar pedicle screw placement: Technical note. J Neurosurg Spine. 2003;99(3):324-9 [41].	5	211	10.55
33	Nolte LP, Visarius H, Arm E, Langlotz F, Schwarzenbach O, Zamorano L. Computer-aided fixation of spinal implants. Comput Aided Surg. 1995;1(2):88-93 [42].	5	201	7.18
34	Gebhard FT, Kraus MD, Schneider E, Liener UC, Kinzl L, Arand M. Does computer-assisted spine surgery reduce intraoperative radiation doses? Spine (Phila Pa 1976). 2006;31(17):2024-7 [43].	4	197	11.59
35	Aoude AA, Fortin M, Figueiredo R, Jarzem P, Ouellet J, Weber MH. Methods to determine pedicle screw placement accuracy in spine surgery: a systematic review. Eur Spine J. 2015;24(5):990-1004 [44].	1	196	24.50
36	Nolte L-P, Zamorano L, Visarius H, Berlemann U, Langlotz F, Arm E, et al. Clinical evaluation of a system for precision enhancement in spine surgery. Clin Biomech (Bristol, Avon). 1995;10(6):293-303 [45].	5	194	6.93
37	Ishikawa Y, Kanemura T, Yoshida G, Matsumoto A, Ito Z, Tauchi R, et al. Intraoperative, full-rotation, three-dimensional image (O-arm)-based navigation system for cervical pedicle screw insertion: Clinical article. J Neurosurg Spine. 2011;15(5):472-8 [46].	4	193	16.08
38	Schlenzka D, Laine T, Lund T. Computer-assisted spine surgery. Eur Spine J. 2000;9(S1):S057-64 [47].	5	188	8.17
39	Nottmeier EW, Seemer W, Young PM. Placement of thoracolumbar pedicle screws using three-dimensional image guidance: experience in a large patient cohort: Clinical article. J Neurosurg Spine. 2009;10(1):33-9 [48].	4	187	13.36
40	Glossop ND, Hu RW, Randle JA. Computer-aided pedicle screw placement using frameless stereotaxis. Spine (Phila Pa 1976). 1996;21(17):2026-34 [49].	5	180	6.67
41	Villard J, Ryang Y-M, Demetriades AK, Reinke A, Behr M, Preuss A, et al. Radiation exposure to the surgeon and the patient during posterior lumbar spinal instrumentation: A prospective randomized comparison of navigated versus non-navigated freehand techniques. Spine (Phila Pa 1976). 2014;39(13):1004-9 [50].	2	171	19.00
42	Rahmathulla G, Nottmeier EW, Pirris SM, Deen HG, Pichelmann MA. Intraoperative image-guided spinal navigation: technical pitfalls and their avoidance. Neurosurg Focus. 2014;36(3):E3 [51].	5	168	18.67
43	Waschke A, Walter J, Duenisch P, Reichart R, Kalff R, Ewald C. CT-navigation versus fluoroscopy-guided placement of pedicle screws at the thoracolumbar spine: single center experience of 4,500 screws. Eur Spine J. 2013;22(3):654-60 [52].	3	167	16.70
44	Mendelsohn D, Strelzow J, Dea N, Ford NL, Batke J, Pennington A, et al. Patient and surgeon radiation exposure during spinal instrumentation using intraoperative computed tomography-based navigation. Spine J. 2016;16(3):343-54 [53].	3	162	23.14
45	Hott JS, Deshmukh VR, Klopfenstein JD, Sonntag VKH, Dickman CA, Spetzler RF, et al. Intraoperative Iso-C C-arm navigation in craniospinal surgery: The first 60 cases. Neurosurgery. 2004;54(5):1131-7 [54].	4	158	8.32
46	Gebhard F, Weidner A, Liener UC, Stöckle U, Arand M. Navigation at the spine. Injury. 2004;35(1):35-45 [55].	5	156	8.21
47	Tabaraee E, Gibson AG, Karahalios DG, Potts EA, Mobasser J-P, Burch S. Intraoperative cone beam-computed tomography with navigation (O-ARM) versus conventional fluoroscopy (C-ARM): A cadaveric study comparing accuracy, efficiency, and safety for spinal instrumentation. Spine (Phila Pa 1976). 2013;38(22):1953-8 [56].	5	154	15.40
48	Ito Y, Sugimoto Y, Tomioka M, Hasegawa Y, Nakago K, Yagata Y. Clinical accuracy of 3D fluoroscopy-assisted cervical pedicle screw insertion: Clinical article. J Neurosurg Spine. 2008;9(5):450-3 [57].	4	154	10.27
49	Merloz P, Tonetti J, Pittet L, Coulomb M, Lavallée S, Troccaz J, et al. Computer-assisted spine surgery. Comput Aided Surg. 1998;3(6):297-305 [58].	2	154	6.16
50	Kamimura M, Ebara S, Itoh H, Tateiwa Y, Kinoshita T, Takaoka K. Accurate pedicle screw insertion under the control of a computer-assisted image guiding system: Laboratory test and clinical study. J Orthop Sci. 1999;4(3):197-206 [59].	4	152	6.33
51	Abe Y, Sato S, Kato K, Hyakumachi T, Yanagibashi Y, Ito M, et al. A novel 3D guidance system using augmented reality for percutaneous vertebroplasty: Technical note. J Neurosurg Spine. 2013;19(4):492-501 [60].	4	149	14.90
52	Elmi-Terander A, Burström G, Nachabe R, Skulason H, Pedersen K, Fagerlund M, et al. Pedicle screw placement using augmented reality surgical navigation with intraoperative 3D imaging: A first in-human prospective cohort study. Spine (Phila Pa 1976). 2019;44(7):517-25 [61].	3	148	37.00
53	Oertel MF, Hobart J, Stein M, Schreiber V, Scharbrodt W. Clinical and methodological precision of spinal navigation assisted by 3D intraoperative O-arm radiographic imaging: Technical note. J Neurosurg Spine. 2011;14(4):532-6 [62].	4	148	12.33
54	Assaker R, Reyns N, Vinchon M, Demondion X, Louis E. Transpedicular screw placement: Image-guided versus lateral-view fluoroscopy:In vitro simulation. Spine (Phila Pa 1976). 2001;26(19):2160-4 [63].	5	148	6.73
55	Kochanski RB, Lombardi JM, Laratta JL, Lehman RA, O’Toole JE. Image-guided navigation and robotics in spine surgery. Neurosurgery. 2019;84(6):1179-89 [64].	4	147	36.75
56	Tormenti MJ, Kostov DB, Gardner PA, Kanter AS, Spiro RM, Okonkwo DO. Intraoperative computed tomography image-guided navigation for posterior thoracolumbar spinal instrumentation in spinal deformity surgery. Neurosurg Focus. 2010;28(3):E11 [65].	3	147	11.31
57	Choi WW, Green BA, Levi ADO. Computer-assisted fluoroscopic targeting system for pedicle screw insertion. Neurosurgery. 2000;47(4):872-8 [66].	5	147	6.39
58	Nolte LP, Slomczykowski MA, Berlemann U, Strauss MJ, Hofstetter R, Schlenzka D, et al. A new approach to computer-aided spine surgery: fluoroscopy-based surgical navigation. Eur Spine J. 2000;9 Suppl 1:S78-88 [67].	5	143	6.22
59	Larson AN, Santos ERG, Polly DW Jr, Ledonio CGT, Sembrano JN, Mielke CH, et al. Pediatric pedicle screw placement using intraoperative computed tomography and 3-dimensional image-guided navigation. Spine (Phila Pa 1976). 2012;37(3):E188-94 [68].	4	142	12.91
60	Holly LT, Foley KT. Percutaneous placement of posterior cervical screws using three-dimensional fluoroscopy. Spine (Phila Pa 1976). 2006;31(5):536-40 [69].	5	142	8.35
61	Kim KD, Patrick Johnson J, Bloch O, Masciopinto JE. Computer-assisted thoracic pedicle screw placement: An in vitro feasibility study. Spine (Phila Pa 1976). 2001;26(4):360-4 [70].	5	142	6.45
62	Scheufler K-M, Franke J, Eckardt A, Dohmen H. Accuracy of image-guided pedicle screw placement using intraoperative computed tomography-based navigation with automated referencing. Part II: Thoracolumbar spine. Neurosurgery. 2011;69(6):1307-16 [71].	4	140	11.67
63	Scheufler K-M, Franke J, Eckardt A, Dohmen H. Accuracy of image-guided pedicle screw placement using intraoperative computed tomography-based navigation with automated referencing, part I: cervicothoracic spine. Neurosurgery. 2011;69(4):782-95; discussion 795 [72].	4	140	11.67
64	Gautier E, Bächler R, Heini PF, Nolte LP. Accuracy of computer-guided screw fixation of the sacroiliac joint. Clin Orthop Relat Res. 2001;393:310-7 [73].	5	139	6.32
65	Yoon JW, Chen RE, Kim EJ, Akinduro OO, Kerezoudis P, Han PK, et al. Augmented reality for the surgeon: Systematic review. Int J Med Robot. 2018;14(4):e1914 [74].	1	138	27.60
66	Schafer S, Nithiananthan S, Mirota DJ, Uneri A, Stayman JW, Zbijewski W, et al. Mobile C-arm cone-beam CT for guidance of spine surgery: Image quality, radiation dose, and integration with interventional guidance: Mobile c-arm CBCT in spine surgery. Med Phys. 2011;38(8):4563-74 [75].	4	137	11.42
67	Ughwanogho E, Patel NM, Baldwin KD, Sampson NR, Flynn JM. Computed tomography-guided navigation of thoracic pedicle screws for adolescent idiopathic scoliosis results in more accurate placement and less screw removal. Spine (Phila Pa 1976). 2012;37(8):E473-8 [76].	4	135	12.27
68	Ishikawa Y, Kanemura T, Yoshida G, Ito Z, Muramoto A, Ohno S. Clinical accuracy of three-dimensional fluoroscopy-based computer-assisted cervical pedicle screw placement: a retrospective comparative study of conventional versus computer-assisted cervical pedicle screw placement: Clinical article. J Neurosurg Spine. 2010;13(5):606-11 [77].	3	134	10.31
69	Merloz P, Troccaz J, Vouaillat H, Vasile C, Tonetti J, Eid A, et al. Fluoroscopy-based navigation system in spine surgery. Proc Inst Mech Eng H. 2007;221(7):813-20 [78].	2	133	8.31
70	Elmi-Terander A, Skulason H, Söderman M, Racadio J, Homan R, Babic D, et al. Surgical navigation technology based on augmented reality and integrated 3D intraoperative imaging: A spine cadaveric feasibility and accuracy study. Spine (Phila Pa 1976). 2016;41(21):E1303-11 [79].	5	130	18.57
71	Nakashima H, Sato K, Ando T, Inoh H, Nakamura H. Comparison of the percutaneous screw placement precision of isocentric C-arm 3-dimensional fluoroscopy-navigated pedicle screw implantation and conventional fluoroscopy method with minimally invasive surgery. J Spinal Disord Tech. 2009;22(7):468-72 [80].	3	129	9.21
72	Liebmann F, Roner S, von Atzigen M, Scaramuzza D, Sutter R, Snedeker J, et al. Pedicle screw navigation using surface digitization on the Microsoft HoloLens. Int J Comput Assist Radiol Surg. 2019;14(7):1157-65 [81].	5	128	32.00
73	Ravi B, Zahrai A, Rampersaud R. Clinical accuracy of computer-assisted two-dimensional fluoroscopy for the percutaneous placement of lumbosacral pedicle screws. Spine (Phila Pa 1976). 2011;36(1):84-91 [82].	4	127	10.58
74	Acosta FL Jr, Thompson TL, Campbell S, Weinstein PR, Ames CP. Use of intraoperative isocentric C-arm 3D fluoroscopy for sextant percutaneous pedicle screw placement: case report and review of the literature. Spine J. 2005;5(3):339-43 [83].	4	126	7.00
75	Puvanesarajah V. Techniques and accuracy of thoracolumbar pedicle screw placement. World J Orthop. 2014;5(2):112 [84].	4	125	13.89
76	Zausinger S, Scheder B, Uhl E, Heigl T, Morhard D, Tonn J-C. Intraoperative computed tomography with integrated navigation system in spinal stabilizations. Spine (Phila Pa 1976). 2009;34(26):2919-26 [85].	3	124	8.86
77	Kraus MD, Krischak G, Keppler P, Gebhard FT, Schuetz UHW. Can computer-assisted surgery reduce the effective dose for spinal fusion and sacroiliac screw insertion? Clin Orthop Relat Res. 2010;468(9):2419-29 [86].	2	123	9.46
78	Smith HE, Yuan PS, Sasso R, Papadopolous S, Vaccaro AR. An evaluation of image-guided technologies in the placement of percutaneous iliosacral screws. Spine (Phila Pa 1976). 2006;31(2):234-8 [87].	5	123	7.24
79	Girardi FP, Cammisa FP Jr, Sandhu HS, Alvarez L. The placement of lumbar pedicle screws using computerised stereotactic guidance. J Bone Joint Surg Br. 1999;81(5):825-9 [88].	4	122	5.08
80	Larson AN, Polly DW, Guidera KJ, Mielke CH, Santos ERG, Ledonio CGT, et al. The accuracy of navigation and 3D image-guided placement for the placement of pedicle screws in congenital spine deformity. J Pediatr Orthop. 2012;32(6):e23-9 [89].	4	121	11.00
81	Dea N, Fisher CG, Batke J, Strelzow J, Mendelsohn D, Paquette SJ, et al. Economic evaluation comparing intraoperative cone beam CT-based navigation and conventional fluoroscopy for the placement of spinal pedicle screws: a patient-level data cost-effectiveness analysis. Spine J. 2016;16(1):23-31 [90].	3	119	17.00
82	Luther N, Iorgulescu JB, Geannette C, Gebhard H, Saleh T, Tsiouris AJ, et al. Comparison of navigated versus non-navigated pedicle screw placement in 260 patients and 1434 screws: Screw accuracy, screw size, and the complexity of surgery. J Spinal Disord Tech. 2015;28(5):E298-303 [91].	3	119	14.88
83	Costa F, Cardia A, Ortolina A, Fabio G, Zerbi A, Fornari M. Spinal navigation: Standard preoperative versus intraoperative computed tomography data set acquisition for computer-guidance system: Radiological and Clinical Study in 100 Consecutive Patients. Spine (Phila Pa 1976). 2011;36(24):2094-8 [92].	3	118	9.83
84	Hott JS, Papadopoulos SM, Theodore N, Dickman CA, Sonntag VKH. Intraoperative Iso-C C-arm navigation in cervical spinal surgery: review of the first 52 cases. Spine (Phila Pa 1976). 2004;29(24):2856-60 [93].	2	118	6.21
85	Laudato PA, Pierzchala K, Schizas C. Pedicle screw insertion accuracy using O-arm, robotic guidance, or freehand technique: A comparative study. Spine (Phila Pa 1976). 2018;43(6):E373-8 [94].	3	117	23.40
86	Hart RA, Hansen BL, Shea M, Hsu F, Anderson GJ. Pedicle screw placement in the thoracic spine: A comparison of image-guided and manual techniques in cadavers. Spine (Phila Pa 1976). 2005;30(12):E326-31 [95].	5	116	6.44
87	Richter M, Amiot L-P, Neller S, Kluger P, Puhl W. Computer-assisted surgery in posterior instrumentation of the cervical spine: an in-vitro feasibility study. Eur Spine J. 2000;9(S1):S065-70 [96].	5	113	4.91
88	Houten JK, Nasser R, Baxi N. Clinical assessment of percutaneous lumbar pedicle screw placement using the O-arm multidimensional surgical imaging system. Neurosurgery. 2012;70(4):990-5 [97].	3	112	10.18
89	Kim TT, Drazin D, Shweikeh F, Pashman R, Johnson JP. Clinical and radiographic outcomes of minimally invasive percutaneous pedicle screw placement with intraoperative CT (O-arm) image guidance navigation. Neurosurg Focus. 2014;36(3):E1 [98].	4	110	12.22
90	Sakai Y, Matsuyama Y, Nakamura H, Katayama Y, Imagama S, Ito Z, et al. Segmental pedicle screwing for idiopathic scoliosis using computer-assisted surgery. J Spinal Disord Tech. 2008;21(3):181-6 [99].	3	110	7.33
91	Uhl E, Zausinger S, Morhard D, Heigl T, Scheder B, Rachinger W, et al. Intraoperative computed tomography with integrated navigation system in a multidisciplinary operating suite. Oper Neurosurg (Hagerstown). 2009;64(5):ons231-40 [100].	4	109	7.79
92	Holly LT, Foley KT. Image guidance in spine surgery. Orthop Clin North Am. 2007;38(3):451-61 [101].	4	109	6.81
93	Kamimura M, Ebara S, Itoh H, Tateiwa Y, Kinoshita T, Takaoka K. Cervical pedicle screw insertion: Assessment of safety and accuracy with computer-assisted image guidance. J Spinal Disord. 2000;13(3):218-24 [102].	5	109	4.74
94	Elmi-Terander A, Nachabe R, Skulason H, Pedersen K, Söderman M, Racadio J, et al. Feasibility and accuracy of thoracolumbar minimally invasive pedicle screw placement with augmented reality navigation technology. Spine (Phila Pa 1976). 2018;43(14):1018-23 [103].	5	108	21.60
95	Rodriguez A, Neal MT, Liu A, Somasundaram A, Hsu W, Branch CL. Novel placement of cortical bone trajectory screws in previously instrumented pedicles for adjacent-segment lumbar disease using CT image-guided navigation: Technical note. Neurosurg Focus. 2014;36(3):E9 [104].	4	106	11.78
96	Allam Y, Silbermann J, Riese F, Greiner-Perth R. Computer tomography assessment of pedicle screw placement in thoracic spine: comparison between free hand and a generic 3D-based navigation techniques. Eur Spine J. 2013;22(3):648-53 [105].	2	103	10.30
97	Perdomo-Pantoja A, Ishida W, Zygourakis C, Holmes C, Iyer RR, Cottrill E, et al. Accuracy of current techniques for placement of pedicle screws in the spine: A comprehensive systematic review and meta-analysis of 51,161 screws. World Neurosurg. 2019;126:664-678.e3 [106].	1	101	25.25
98	Ohba T, Ebata S, Fujita K, Sato H, Haro H. Percutaneous pedicle screw placements: accuracy and rates of cranial facet joint violation using conventional fluoroscopy compared with intraoperative three-dimensional computed tomography computer navigation. Eur Spine J. 2016;25(6):1775-80 [107].	4	87	12.43
99	Du JP, Fan Y, Wu QN, Wang DH, Zhang J, Hao DJ. Accuracy of pedicle screw insertion among 3 image-guided navigation systems: Systematic review and meta-analysis. World Neurosurg. 2018;109:24-30 [108].	3	87	17.40
100	Tajsic T, Patel K, Farmer R, Mannion RJ, Trivedi RA. Spinal navigation for minimally invasive thoracic and lumbosacral spine fixation: implications for radiation exposure, operative time, and accuracy of pedicle screw placement. Eur Spine J. 2018;27(8):1918-24 [109].	4	76	15.20

The observed increase in mean citations per year in recent decades suggests a growing impact and visibility of scholarly works. Factors such as improved communication technology, increased collaboration, and the rise of open-access initiatives contribute to this trend. However, it's crucial to consider that higher citation rates don't necessarily reflect improved research quality and may be influenced by various factors.

The article ranked first on the list was “Accuracy of pedicle screw insertion with and without computer assistance: a randomized controlled clinical study in 100 consecutive patients” by Laine T et al., published in the *European Spine Journal *[10]. The second most cited article was “Pedicle screw placement accuracy: A meta-analysis” by Kosmopoulos V and Schizas C, published in 2007 in *Spine (Phila Pa 1976)* [11]. The third-ranked article on the list, “Accuracy of pedicle screw placement: a systematic review of prospective in vivo studies comparing free hand, fluoroscopy guidance, and navigation techniques,” was written by Gelalis ID et al. and issued in the *European Spine Journal* in 2012 [12].

The oldest article recorded in the list, “A technique for accurate transpedicular screw fixation using CT data and a 3-D optical localizer,” was published in January 1995 by Lavallée S et al. in the *Computer Aided Surgery Journal* [27]. The most recent article is “Pedicle screw navigation using surface digitization on the Microsoft HoloLens” by Liebmann F et al. (2019) published in the *International Journal of Computer Assisted Radiology and Surgery* [81].

The top 100 most cited publications were published in a total of 24 journals, 64% of them published within the top four, being *Spine* was the most productive one, with 33 articles, followed by *European Spine Journal *with 15 articles and the third and fourth most frequent were the *Journal of Neurosurgery: Spine and Neurosurgery* with eight articles both. The rest of the journals published four or fewer articles. The Impact Factor for the top four journals was found to be in the first quartile within their category (Table 3).

**Table 3:** Top journals of publication * Four-year impact factor Scimago Journal and country rank until the year 2022 ** Four-year impact factor Scimago Journal and country rank until the year 2019

Journals	Impact factor*	Number of articles
Spine	3.22	33
European Spine Journal	3.01	15
Journal of Neurosurgery: Spine	3.02	8
Neurosurgery	2.50	8
Neurosurgical Focus	3.74	4
Journal of Spinal Disorders and Techniques	2.58**	4
The Spine Journal	4.15	4
Computer Aided Surgery	NA	3
World Neurosurgery	1.91	3
Clinical Neurosurgery	1.95	2
Clinical Orthopaedics and Related Research	2.07	2
Journal of Neurosurgery	3.44	2
Orthopedic Clinics of North America	2.63	1
Clinical Biomechanics	2.30	1
Injury	2.66	1
International Journal of Computer Assisted Radiology and Surgery	3.89	1
International Journal of Medical Robotics and Computer Assisted Surgery	3.03	1
International Orthopaedics	3.05	1
Journal of Bone and Joint Surgery - Series B	3.11	1
Journal of Orthopaedic Science	1.69	1
Journal of Pediatric Orthopaedics	2.18	1
Medical Physics	4.05	1
Proceedings of the Institution of Mechanical Engineers, Part H: Journal of Engineering in Medicine	2.23	1
World Journal of Orthopedics	2.54	1

**Figure 4:**
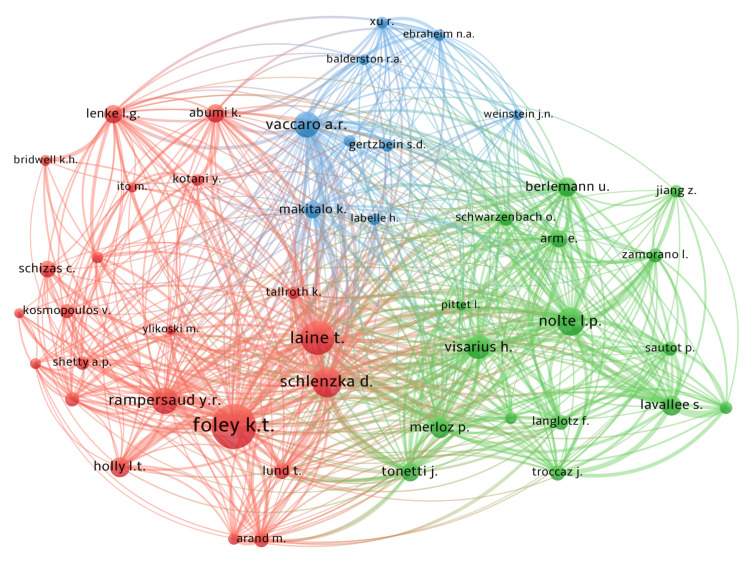
Visualization of the co-citation network patterns among authors regarding navigation in spine surgery Each circle represents an author, with the size of the circle indicating the number of times that author has been cited; the larger the circle, the more citations the author has received. Each color (green, red, and blue) represents a distinct group of co-cited authors, illustrating clusters of authors who are frequently cited together, and suggesting thematic or collaborative similarities among authors. Lines connecting the circles denote co-citation relationships between individual authors, highlighting the strength and frequency with which authors are cited together in the literature.

There were 22 authors who contributed with three or more publications to the top 100 cited list (Table 4). The authors with the most first authorships were Langston T. Holly and Lutz P. Nolte both with four articles (773 citations and 953 citations, respectively), while the author with the highest count of articles was Lutz P. Nolte, with six articles (1,491 citations). Other prominent authors were Foley KT, Laine T, Schlenzka D, and Holly LT. A visual representation of the author's co-citation patterns is shown in Figure 4.

**Table 4:** Top authors in the 100 most cited papers

Authors	Number of articles
Nolte LP	6
Foley KT	5
Schlenzka D	4
Laine T	4
Holly LT	4
Rampersaud YR	3
Zamorano LJ	3
Visarius H	3
Troccaz J	3
Skulason H	3
Richter M	3
Papadopoulos SM	3
Nachabe R	3
Merloz P	3
Lund T	3
Langlotz F	3
Härtl R	3
Gebhard FT	3
Elmi-Terander A	3
Berlemann U	3
Babic D	3
Amiot LP	3

**Figure 5:**
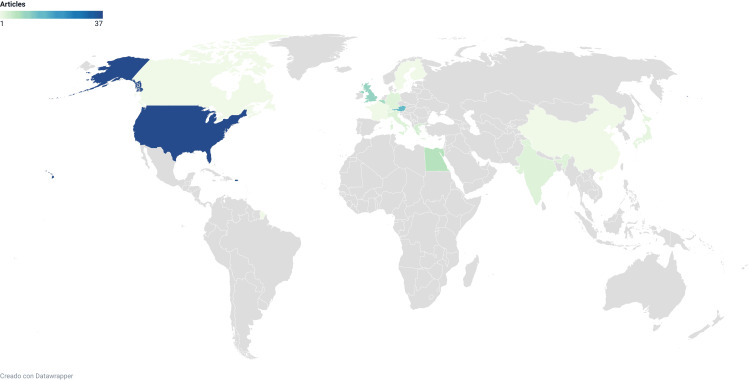
Top countries of origin of articles based on first author affiliation

**Figure 6:**
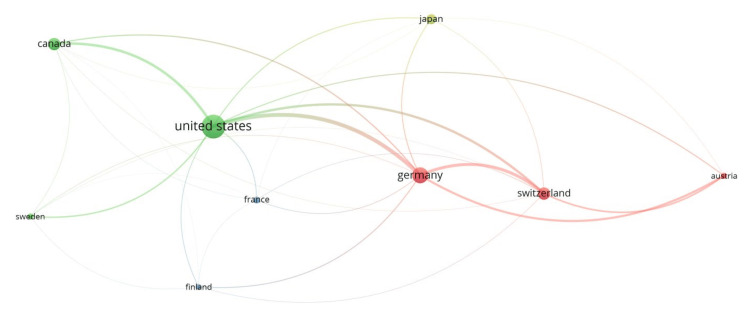
Visual representation of the international collaboration landscape for articles from different countries Each circle represents a country, with the size of the circle corresponding to the number of articles produced by that country; the larger the circle, the more articles published. Colors indicate countries that frequently collaborate with each other, forming distinct groups of international partnerships. Lines connecting the circles denote collaborative efforts between countries, with the thickness of the lines reflecting the strength and frequency of these collaborations.

**Figure 7:**
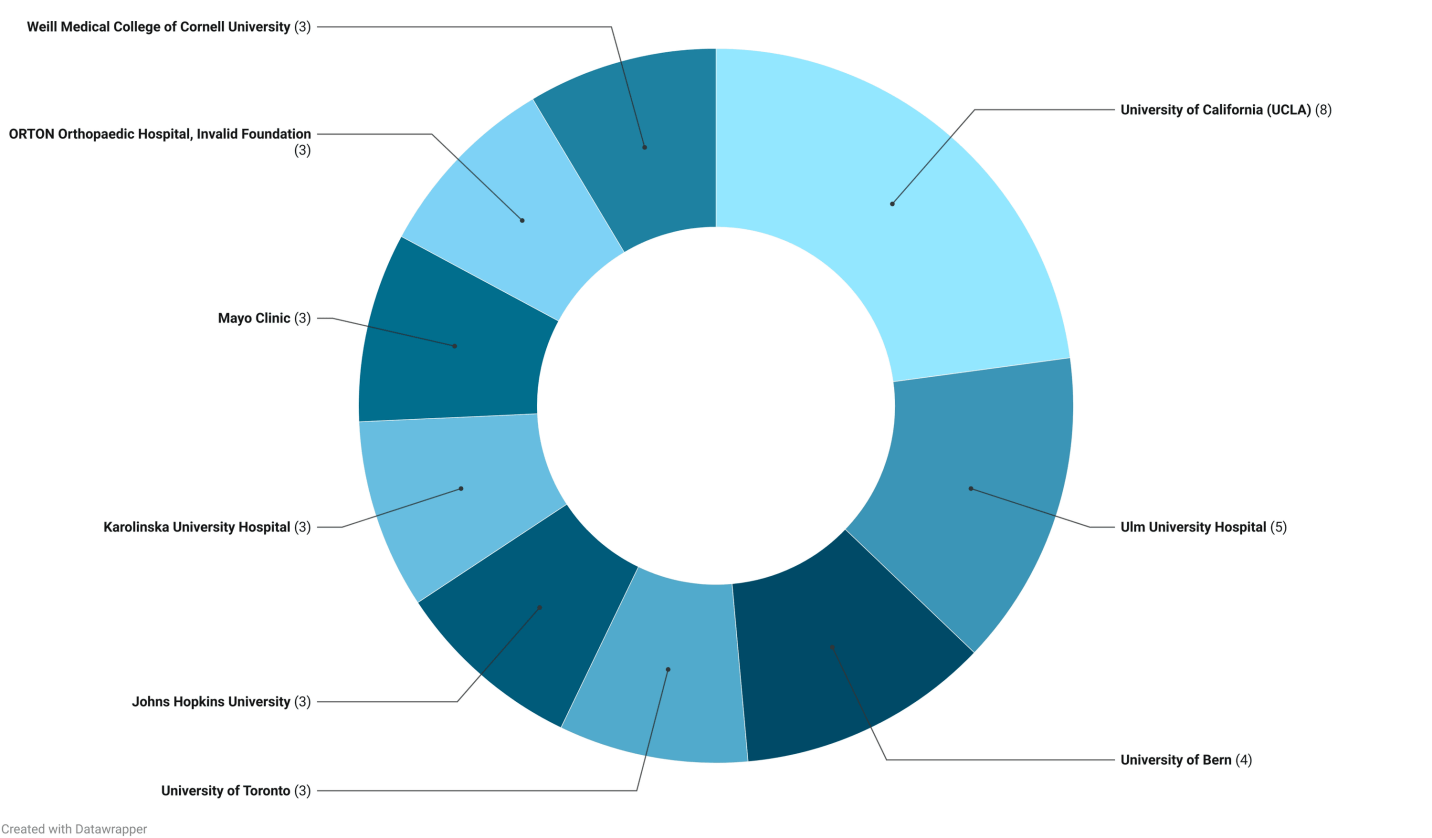
Top institutions of origin of articles based on first author affiliation

Articles originated from 16 countries, with the top 5 being in the list the United States with 37 publications, Germany with 15 publications, Japan with 10 publications, Switzerland with nine publications, and Canada with seven publications (Figure 5). Figure 6 shows the strongest collaboration patterns between countries. Additionally, the leading institutions for the 100 most cited articles were found to be the University of California with eight articles, Ulm University with five articles, and the University of Bern with four articles. Other institutions such as the University of Toronto, Johns Hopkins University, Karolinska University Hospital, Mayo Clinic, Orton Orthopaedic Hospital, and Weill Medical College contributed three articles each (Figure 7).

**Figure 8:**
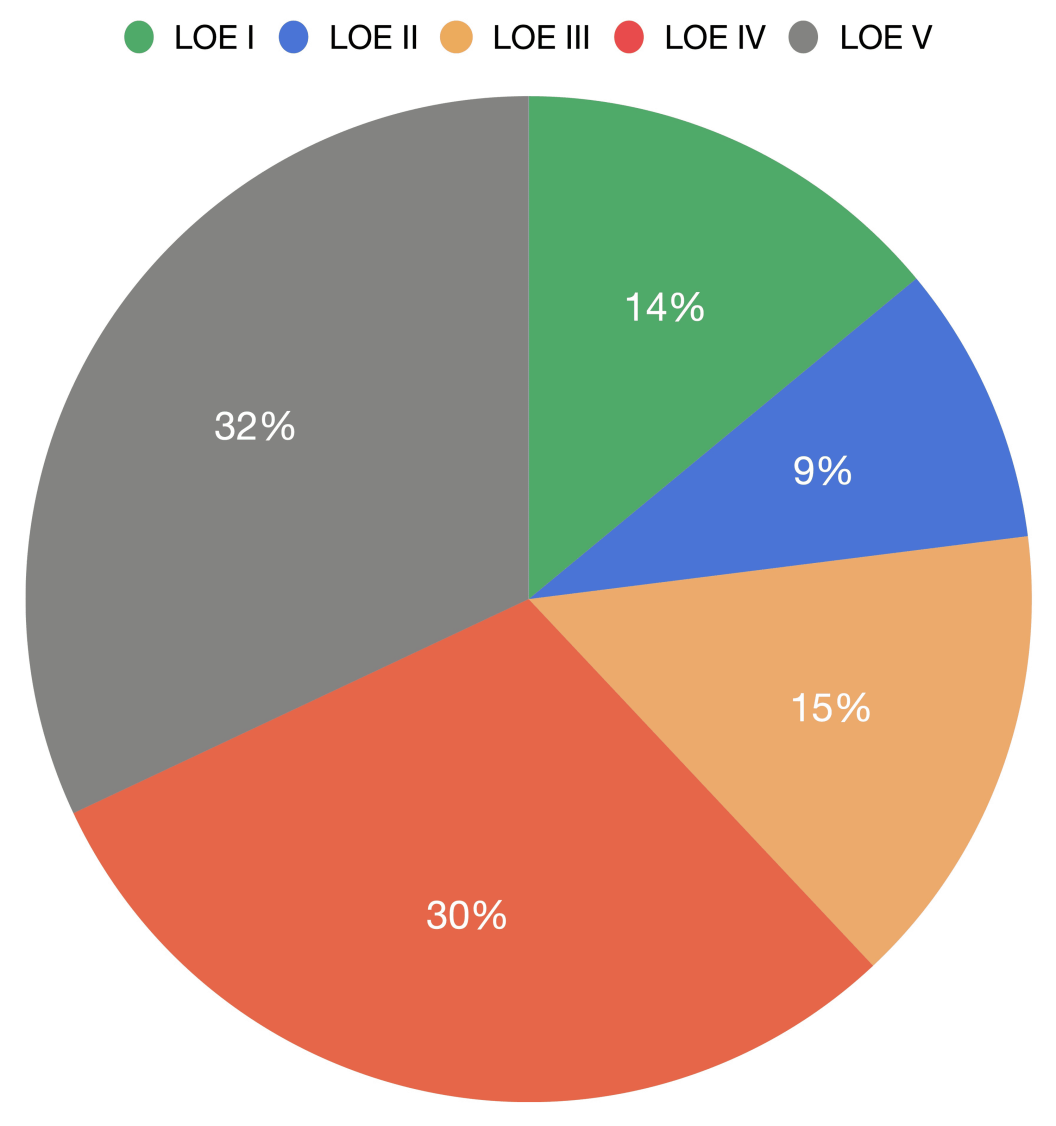
Level of evidence (LOE) distribution for all articles

The most common type of publication was clinical research (73%) and then clinical reviews (21%); among these, the most frequent area was clinical outcomes (54 articles), then meta-analysis and systematic reviews with 11 articles. Interestingly there were only four articles regarding RCTs [110]. According to the LOE, the most common was level V with 32 articles, meanwhile, evidence levels I and II represented 23% of the list (Figure 8).

Discussions

This study has shed light on the most cited papers for navigation in spine surgery and creates a list of essential research published over the last three decades. We conducted a thorough examination of the distinctive attributes inherent to each article, highlighting the authors, journals, and countries that play pivotal roles as platforms and promoters for the widespread dissemination of evidence in the field of spinal surgery.

The top cited article of the list, by Laine T et al. (2000), assesses the accuracy of computer-assisted versus conventional screw placement under clinical conditions in 100 consecutive patients [10]. In the trial, they found a significant increment in the rate of pedicle perforations in the conventional technique group compared to the computer-assisted group (13.4% vs 4.6%), especially through the medial wall. The use of navigation is especially helpful compared to non-navigated techniques, in cases of spine deformity surgery because it significantly reduces pedicle breaches (23% vs. 2%) as well as surgical time and radiation dosage [14,19].

The second most cited paper, by Kosmopoulos and Schizas (2007), analyzes 130 studies from 1966 until 2006 to find the accuracy of pedicle screw placement with different techniques. For the in vivo population, they found a median accuracy of 95.1% vs 90.3% in 3,059 screws with navigation and 12,299 screws without navigation, respectively. The overall accuracy for in vivo and cadaveric placement of pedicle screws with and without navigation was 91.3% (37,337 screws) [11].

The third-ranked article, by Gelalis ID et al. (2012), includes 26 prospective in vivo studies (1,105 patients and 6,617 screws) that compare the accuracy of pedicle screw position between three techniques. They found a significantly lower screw malposition rate in the navigation-assisted group (0-11%) compared with freehand (6-31%) and fluoroscopy based (15-72%) techniques [12]. Navigation has been demonstrated to decrease the length of hospital stay, blood loss, and proportion of screws with the cortical breach (navigated 63.4% vs. freehand 30.6%, p < 0.0001) while increasing the clinical accuracy of screws position (navigated 93.9% vs. freehand 89.6%, p < 0.05) [111].

The oldest cited article in the list, by Lavallée S et al. (1995), describes a computer-assisted spine surgery system developed by his team in 1985, validated with in vitro experiments, and later used in seven patients obtaining positive results [27]. The navigation system is similar to what Nolte et al. (1995) described, while the former uses multiple software-generated reference points in the vertebrae; the latter uses a single point reference point to create a 3D-dimensional image of the vertebrae, both from preoperative CT scans [42].

As for the most recent article, published by Liebmann F et al. (2019) in July 2019 with the aim to develop a novel navigation technique that combines a surgical navigation system with virtual reality (Microsoft HoloLens), pedicular screws were inserted in 3D printed lumbar spine phantoms and evaluated through postoperative CT scans. Results are promising, with an approximate time for surface digitalization of 125 seconds, and a mean error for screw trajectory orientation and insertion points of 3.38±1.73 degrees and 2.77±1.46 mm, respectively [81].

The largest number of articles were published in the journal *Spine* (33%), and countries with the highest research output were the United States (37%), Germany (15%), Japan (10%), Switzerland (9%) and Canada (7%), similar to what other orthopedic and spine bibliometric analyses have shown [112-114].

Technology has improved the healthcare system over the past decades, especially in the field of spine surgery; the development of navigation has increased accuracy and safety for patients and medical staff. Screw pedicle instrumentation has been the main focus, but its utility can be applied to complex deformity cases and spinal tumor surgery while decreasing radiation exposure to surgeons [32,115,116].

In the decade from 1990 to 1999, the primary topics were feasibility and in vitro studies, with only a few clinical studies, overall, with a low grade of evidence. From 2000 to 2009, the main focus was reviews and retrospective clinical outcome studies, level of evidence improved and humanistic outcome studies such as radiation exposure, were important research topics. In the last decade from 2010 to 2019, systematic reviews and meta-analysis accounted for a large part of the literature, further increasing the level of evidence compared to previous dates. The emergence of navigation integrated with technology such as head displays for virtual reality was noted, especially in the most recent articles.

Dea N et al. (2016) performed an economic evaluation for intraoperative 3D imaging paired with a navigation system; it showed a cost-effectiveness ratio of $15,961 per reoperation avoided (6-8%) compared to conventional fluoroscopy (0-2%), being economically justified for hospitals with 250 or more spinal surgeries per year [90].

There are several limitations in this study. The first is that a ranking based on citation count does not entirely correlate to the importance and impact of the articles, but it is an objective way to stratify their contribution to the field. Another limitation was that the volume of publications per year for each journal was not accounted for, possibly creating a bias toward journals that publish bi-weekly rather than bi-monthly. Lastly, our biggest limitation is the existence of multiple navigation systems and related terms to describe them, generating ambiguity in the terminology and creating a possible bias against “classical” papers.

## Conclusions

The bibliometric analysis of the 100 most cited articles on intraoperative image-guided navigation in spine surgery reveals significant advancements and increased acceptance of navigation technologies over the past three decades. This study, the first of its kind, highlights the positive growth of research since the 1990s and underscores the need for higher-level evidence in the literature. The last decade has witnessed a surge in high-quality publications, driven by the integration of emerging technologies such as augmented and virtual reality, which have significantly enhanced surgical precision, reduced complication rates, and improved patient outcomes. The high citation counts observed in certain articles can be attributed to several factors, including methodological rigor, relevance to ongoing debates, and innovative approaches introduced by these studies. For example, articles that address critical issues in spine surgery with robust clinical data or comprehensive meta-analyses tend to attract more citations. The influence of high-impact journals like *Spine* and the *European Spine Journal *also plays a crucial role in disseminating widely cited research, as articles published in these journals reach a larger and more diverse audience.

However, it is important to recognize the limitations of using citation counts as the sole measure of an article's impact. Citation disparities can arise due to factors such as the age of the article, with older publications having had more time to accumulate citations, or the predominance of English-language journals, which may lead to the underrepresentation of research published in non-English journals. Furthermore, citation counts may not fully capture the quality or innovation of a study, as they can be influenced by the popularity of the topic at the time of publication or the network of the authors. The findings of this study emphasize the importance of continuing to produce high-quality, methodologically sound research that is relevant to ongoing debates in the field of spine surgery. As emerging technologies like virtual reality continue to offer immersive training and precise preoperative planning, their role in enhancing the accuracy and safety of spine surgeries becomes increasingly vital. Future research should focus on generating higher levels of evidence to validate the efficacy and safety of these technologies, ensuring their widespread adoption in clinical practice. This study provides valuable insights for spine surgeons, fellows, and residents, highlighting the most influential research in navigated spine surgery and underscoring the ongoing need for innovation and high-quality evidence in this evolving field.
